# A Survey on Security and Privacy in Emerging Sensor Networks: From Viewpoint of Close-Loop

**DOI:** 10.3390/s16040443

**Published:** 2016-03-26

**Authors:** Lifu Zhang, Heng Zhang

**Affiliations:** 1 Wenzhou Vocational & Technical College, Wenzhou 325000, China; zlifu602@126.com; 2 Huaihai Institute of Technology, Lianyungang 222000, China

**Keywords:** sensor networks, security, privacy, game theory, remote state estimation, feedback control

## Abstract

Nowadays, as the next generation sensor networks, Cyber-Physical Systems (CPSs) refer to the complex networked systems that have both physical subsystems and cyber components, and the information flow between different subsystems and components is across a communication network, which forms a closed-loop. New generation sensor networks are found in a growing number of applications and have received increasing attention from many inter-disciplines. Opportunities and challenges in the design, analysis, verification and validation of sensor networks co-exists, among which security and privacy are two important ingredients. This paper presents a survey on some recent results in the security and privacy aspects of emerging sensor networks from the viewpoint of the closed-loop. This paper also discusses several future research directions under these two umbrellas.

## 1. Introduction

As the next generation sensor networks, Cyber-Physical Systems (CPSs) are the complex systems that consist of cyber elements, physical elements and an interface that connects the different subsystems. The physical subsystems typically evolve according to some physical laws (e.g., Newton’s laws). Their stability, proper functioning and performance guarantee are the main objectives of control. The cyber elements, e.g., a central controller/computing device, carry out desired computations, and the interface governs the information flow between the physical subsystems and the cyber components.

The term CPS appeared five years ago [1] in a report by the President’s Council of Advisors on Science and Technology (PCAST) to the President of the United States, where CPS was listed as the first top technical priority in networking and information technology research and development.

**Figure 1:**
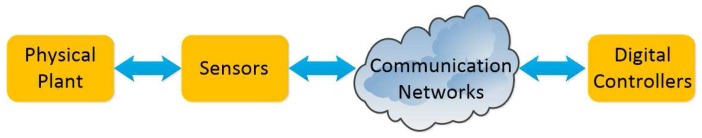
A simple networked control system.

Kim and Kumar in [2] defined a CPS to be an engineering system integrating computing, control and communication, *i.e.*, $C^{3}$. Figure 1 shows a simple example of a CPS, *i.e.*, a networked control system, where the physical plants are the physical subsystems, the digital controllers are the cyber components and the communication network is the interface.

More sophisticated examples than the simple feedback system in Figure 1 include power networks, social networks, smart transportation systems, sensor networks, smart buildings, *etc.* [2,3,4,5,6,7,8,9,10]. These systems are often distributed in nature and have a hierarchical structure. The information flow among the various subsystems is typically asynchronous. The complexity of such systems is enormous and is continuing to grow due to technological advancement and their ever expanding application domains. The National Science Foundation (NSF) [11] of the United States started to support fundamental and applied research inn CPSs in 2008.

The research of CPSs is multi-faceted, from hardware to software, from fundamental theory to real implementation and across many different disciplines. The research activities in CPS have been growing enormously in the past few years, where opportunities and challenges co-exist. This paper focuses on the recent results of two important aspects of CPSs, namely, CPS security and CPS privacy.

In the inchoate study of CPS, secure communication is an important concern for the system designer. Data deficiency, including packet delay, packet dropping and quantizing errors, has been widely investigated. However, if untruthful data, which may be generated by malicious attackers, are received by the system elements for computation and control, a serious incident may occur with incalculable losses. A famous example is that Iran’s nuclear facilities were attacked by Stuxnet in 2010. The research in this field is still very limited.

As the applications of CPSs become pervasive in our daily life (including many national critical infrastructures, such as smart grids and communication systems), enhancing the safety and secure measures of such systems becomes urgent. Existing security measures and approaches are not adequate to address the new challenges introduced by CPSs where information flow and physical systems are tightly coupled together.

Attacks in CPSs can take various different forms, such as inadvertent infiltration through infected devices and network-based intrusion by exploiting poorly-configured firewalls [12]. In case an attacker obtains the privilege to the cyber space and is able to control the desired device, he/she is able to spread malware, compromise sensing and communication equipments and inject false information in measured or computed data. The resulting consequences can be severe, e.g., wide area power blackout in a national power grid and crash and failure of safety-critical infrastructure (nuclear power plants and military facilities). A key question here is how to develop novel algorithms that can identify and detect possible false (or bad) data and isolate the malfunctioning nodes from the network?

Designing a good CPS with privacy consideration is also becoming increasingly important. In many applications, a global objective cannot be achieved without active participation of many individual users. Such a global objective, for example obtaining the average electricity consumption of the households in a small community, may not need the exact electricity usage (private information) of each household, but only need their collective data. On the other hand, the more information the power plant has access to, the better coordination can be arranged, and hence, more detailed electricity pricing information can be predicted and released, which in turn will benefit each household. Thus, by sacrificing minor privacy (or possibly no sacrifice at all via a smart design), a user may significantly increase his/her payoff. Natural questions to ask here are whether the privacy of each individual can be well maintained while at the same time certain global objectives are met, and how to achieve a desired tradeoff between individual’s privacy and payoff.

In this survey paper, we present the works from many different areas that focused on CPSs security and privacy. We will also discuss a few research directions around these two topics, which we think are important in the analysis and design of a secure CPS with privacy guarantee.

In Section 2, we go over some recent results in CPSs security, which studied various different forms of potential attack patterns and security indexes and suggested possible counter measures. In Section 3, we provide an overview of the related works in CPSs privacy, which proposed different privacy notations, measures and strategies to maintain a desired level of privacy. Some concluding remarks and opening questions in CPSs security and privacy are presented at the end.

## 2. Cyber-Physical Systems Security

**Figure 2:**
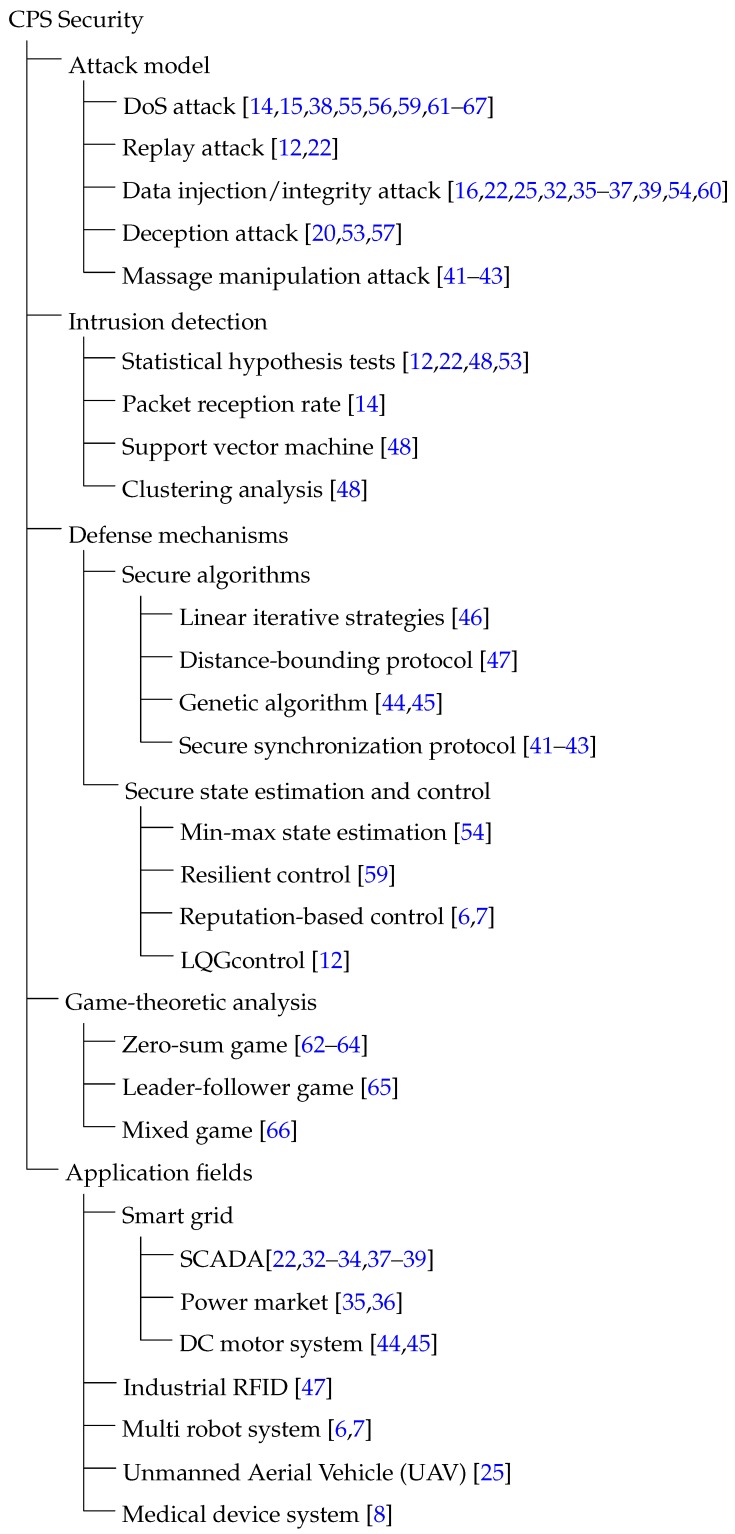
A summary of Cyber-Physical System (CPS) security.

We discuss in this section some of latest achievements in CPS security. In Section 2.1, we look back at some works that studied the general CPSs models with some detailed security analysis. In Section 2.2, we review the latest works on the security of smart grids. In Section 2.3, some achievements for secure algorithms are discussed. In Section 2.4, we present some works that focused on secure networked state estimation and networked control. In Section 2.5, game-theoretical models for addressing CPSs’ security are discussed. We also provide a summary of CPS security in Figure 2 in the end of this section.

### 2.1. Security Analysis of General Cyber-Physical Systems

Researchers have investigated security issues on various kinds of cyber attacks for closed-loop general CPSs. Typical cyber attacks on CPS include Denial-of-Service attacks (DoS) [13,14,15], replay attacks [16,17], data injection/integrity attacks [18,19], deception attacks [20], and so on.

A DoS attacker can prevent the communications between physical elements and cyber elements in a CPS. For example, the cyber attacker can jam the wireless communication channel to degrade the packet reception rate [13]. Zhang *et al*. [14] considered the scenario where a sensor sends its data to a remote estimator through a wireless channel for state estimation, while an attacker decides whether or not to lunch a DoS attack to jam the communication channel at each sampling time. Using both the expected average estimation error and the expected terminal estimation error as performance indices, the authors presented optimal attack schemes, respectively. They also proposed optimal attack strategies to avoid being detected. They further investigated the optimal attack schemes when the sensor and the attacker both have an energy constraint in [15].

The replay attacker records the transmitting data and then repeats the recorded data sequentially. The Stuxnet attack can be modeled as a replay attack [21]. Mo *et al*. [22] studied the effect of replay attacks on a SCADA system. They studied the feasibility of this attacker and proposed the corresponding counter measures to improve the probability of detection. Zhu *et al*. investigated the effect of replay attack on the control performance [23]. This paper considered the scenario that the attacker occupies the communication channel between sender and receiver and then replays the records to replace the real data from the sender. The authors provided a novel feedback control method to reduce the effect of replay attack. The detection of a replay attack is also an interesting issue in the field of CPS security. Mo *et al*. provided a detection method in which an extra Gaussian random value is injected into the data before transmitting [17]. The key idea of this method is to discover the replay attack by sacrificing the cost of system control. Thus, it is necessary to balance the success rate of detection and the control performance. This problem has been solved by Miao *et al*. in [24]. They have presented a stochastic game method to balance the attack detection rate and the optimal control cost.

The data injection attack is implemented by deliberately adding false data to the real information to destroy the performance of CPS. Mo *et al*. [12] discussed some general cyber threats in CPS and provided a few novel countermeasures. They proposed a few system-theoretic approaches to contingency analysis and detection of anomalies in a sensory system. They used a simple linear system as an example, which runs a Kalman filter, an LQGcontroller and a $\chi^{2}$ failure detector and is subject to a potential data integrity attack. They presented a quantitative index of the system resilience by investigating the feasible set of the adversary’s attack strategies without being detected. They also analyzed the corresponding state estimation error under these attacks. In [16], the maximum impact of stealthy bias injection attacks was derived. It was shown that the corresponding optimal policy does not require perfect model and system knowledge. These attack strategies were illustrated and verified experimentally on a quadruple-tank process controlled over a wireless network.

Deception attacks and other types of attacks also have been deeply studied by researchers. A deception attacker can manipulate the sensors and other physical elements in CPS by injecting malicious codes into the programs [20]. Then, the sensors’ transmitting schedules are deliberately falsified, and system performance is deteriorated. Kwon *et al*. [25] analyzed a system’s response under false data injection attacks. They presented three types of stealthy deception attacks in terms of the attacker’s capacity. In accordance with *a priori* knowledge about the system evolution and utilizing existing hypothesis testing algorithms, they derived necessary and sufficient conditions under which the attacker is able to perform each type of attack without being perceived. An Unmanned Aerial Vehicle (UAV) navigation example is given to illustrate the threat of these cyber attacks. Cheminod *et al*. [26] discussed the security issues for a variety of industrial distributed computing systems under general cyber attacks. The authors presented a complete analysis, which provides a satisfactory degree of security for industrial CPS in relation to a few crucial elements that help lower the potential risks below a preset level and acceptable scope. Pasqualetti *et al*. [27] presented a unified modeling framework for cyber-physical systems subject to attacks. They modeled the malicious attacks as unknown inputs, which affect the system state and the sensor measurement data. The concepts of the observability and identifiability of an attack were defined. From a system-theoretic and graph-theoretic angle, the authors provided fundamental monitoring limitations. Then, based on geometric control theory, distributed control and parallel computation, centralized and distributed monitors were designed.

### 2.2. Smart Grid and Power Systems

Smart grid and power systems are important and representative examples of CPSs [28,29,30,31]. Their secure control has been a heated area of research in the past few years.

A Supervisory Control and Data Acquisition (SCADA) system is a typical CPS and is widely used for remote monitoring and control in power systems and smart grids. Several literature works have investigated the security of SCADA. Sandberg *et al*. [32] considered false data injection attacks against remote state estimation using a SCADA system. They provided two security indices, which quantify the smallest number of measurements and the smallest magnitude of the measurement vectors necessary for an attacker to accomplish his or her goals while avoiding the activation of the false-data alarms. The indices are difficult to obtain in closed form, but can be approximated using matrix searching techniques or computed via convex optimization. Queiroz *et al*. [33] constructed a quantification model that provides the service heterogeneity and interdependencies to compute the survivability of a SCADA system. They used network traffic to calculate the information diversity score. The metric of system performance is defined by it. The authors also provided a few novel models to automatically build a Bayesian network, and they adopted the Bayesian network to conclude about the survivability of the SCADA system. Hendrickx *et al*. [34] considered the resilience of a SCADA system for an electric power network under certain types of cyber-attacks. The authors analyzed the vulnerability of the measurement system whose communicated data can be modified by a malicious attacker. The problem was shown to be NP-hard. The authors then showed that this issue with the full measurement is equivalent to a standard Min-Cutproblem and can be done by standard optimization methods.

The security of the power market has also been deeply studied from different viewpoints. Xie *et al*. [35] studied the economic impact of integrity data attacks on power market operations. They showed how malicious attacks can be launched by compromising selected pairs of sensors. They further gave the optimal attacking strategy subject to a limited number of compromised sensing nodes, which is formulated as a convex optimization problem. Numerical examples are illustrated in a real-time IEEE 14-bus system testbed. Tan *et al*. [36] studied the vulnerability of Real-Time Pricing (RTP) when data integrity attacks are present in the smart grid. A control-theoretic method was used to derive the RTP stability conditions under two broad types of integrity attacks: the scaling attack and the delay attack. The authors showed that the RTP system may become unstable only if the scaling attacker can manipulate the price signals by cutting down the values in the smart meter or the delay attacker provides old prices to more than half of all users. Their results provided useful design guidelines for a system administrator to study the effect of potential attacks on system stability, hence taking adequate measures to guarantee the secure operations of the RTP system.

Another research aspect in the security of the power system and smart grids is from the viewpoint of system control and optimization. Kosut *et al*. [37] considered malicious data attacks against state estimation in a smart grid and provided the corresponding counter measures. First, they used a graph-theoretic method to search for the minimal set of meters so that the adversary’s malicious data attacks are unobservable. When the adversary cannot perform an unobservable attack due to meter access limitation, the minimum residue energy attack was proposed to tradeoff the damage caused to the state estimator and the alarm triggering probability. A detector with hypothesis testing was then derived at the controller side. Liu *et al*. [38] investigated the impact of DoS attacks on the load frequency control (LFC) of a smart grid. They modeled the LFC of a power grid as a switched system and pointed out that the DoS attack can result in system instability. They proved that the attack can significantly impact the system performance if the DoS attack is launched before the power system states converge. Sou *et al*. [39] considered attack detection and isolation on a power network using power flow measurements. The authors proposed a novel measurement residual, which can be calculated in real time to isolate the distributed data attack in a large-scale power network. They showed the effectiveness of the proposed scheme under some numerical case studies on an IEEE 14-bus benchmark system.

Renewable energy, which is collected from the sun, wind, waves and other natural resources, is an important part of power networks. Malicious attacks can prominently impact the performance of renewable energy systems [40]. Johansson in [40] discussed the potential risk and its effect on system operation and production benefits in renewable energy systems. He also pointed out that the security issues on renewable energy systems is becoming prominent with its increasing proportion in power systems. As far as we know, the theoretical and technical research works in this field are still very few.

### 2.3. Secure Algorithms

Synchronization protocols, an important component in CPS, are vulnerable to cyber attacks. He *et al*. [41] studied the impact of message manipulation attacks in CPS. They designed a security protocol, a new adaptive parameter test method, to protect the average consensus-based time synchronization protocol. They further designed the Secured Maximum consensus-based Time Synchronization (SMTS) protocol to defend message manipulation attacks [42]. The SMTS protocol can update the clock skew and the offset with information compensation simultaneously. The SMTS protocol consists of six main components, namely message reception and verification, hardware clock checking process, logical clock checking process, updating logical clock based on MTS, message generation and authentication and message broadcasting. Motivated by [42], Zhao *et al*. [43] also investigated secure synchronous consensus against message manipulation attacks. The authors first proposed a Secure Synchronous Consensus Algorithm (SSCA) and proved that the proposed secure algorithm converges exponentially. They also investigated the effect of the exact behavior of message manipulation attack.

Zeng and Chow [44,45] studied performance-security tradeoff optimization for a distributed CPS using the Coevolutionary Genetic Algorithm (CGA). They presented a performance-security tradeoff model with a DC motor system. They further implemented a Simulink-based testbed to illustrate the proposed algorithm, and their results demonstrated that CGA returns the optimal solutions efficiently for a security-performance tradeoff model of a distributed CPS.

The performance of distributed function calculation by a group of interconnected agents in the presence of malicious attackers has been studied by Sundaram and Hadjicostis [46]. Each agent iterates its value by weighted summing of the values from its own and those received from its neighbors via a linear iterative strategy. Malicious attackers, on the other hand, update their values arbitrarily. Their work revealed that the resilience of the linear iterative method against malicious attacks depends on the number of vertex-disjoint paths and that of the malicious attackers.

Distance-bounding, a crucial neighbor false detection approach in accordance with the round trip time of cryptographic challenge-response pairs, cryptographically determines an upper bound of the physical distance between two communicating parties. The paper [47] gave a brief overview of distance-bounding protocols and discussed the possibility of the implementation of such protocols for industrial RFID and real-time location applications. These applications require reliability and real-time communications. The practical resource requirements and performance tradeoffs involved are illustrated using a sample of distance-bounding protocols. Some other research challenges for practical implementation were also discussed.

Detecting a network anomaly is a basic task in the operation of CPS. Wang *et al*. [48] has summarized five approaches for the detection of a network anomaly, including support vector machines, hypothesis tests and the clustering method. They compared the effectiveness of these approaches by simulation and showed the advantages and disadvantages of them. They also pointed out that a combination of these approaches can improve the performance of anomaly detection.

Vollmer and Manic [49] presented a design method for self-configuring honeypots, which passively check the network traffic of CPS and positively adjust to the sensing environment. Six different detection tools in cyber-space were assessed, and Ettercap was provided to identify the host. According to the output of Ettercap XML, the authors developed a new secure algorithm and reformed the framework of Honeyd. The authors investigated the performance of the proposed algorithm on a college network and a sensor network by implementing a collaborative employment scenario.

### 2.4. Secure State Estimation and Control

The quality of state estimation and the control performance for CPS are the main concerns in control society [50,51,52]. They may tremendously be effected by cyber attacks.

Secure state estimation has been studied from the aspects of attack evaluation, intrusion detection and defense strategies. Teixeira *et al*. [53] analyzed the effects of possible deceptions attacks for state estimators. The attacks have limited or out-of-date knowledge of the network and the true system parameters. The authors showed that with a more accurate system model, deception attacks can lead to a more severe impact. The authors also presented conditions that the attacker should possess in order to bypass a bad data detection mechanism. For both linear and nonlinear state estimation, they proposed some policies to synthesize deception attacks. Mo *et al*. [54] considered an integrity attack for remote state estimation, where a binary random variable is estimated based on *m* noisy measurements. The authors assumed that the attacker can access the true value of the states and the value of the measurements, but is only able to modify at most *n* out of these *m* measurements. By solving a min-max problem, they proposed an optimal detector to minimize the “worst case” probability of error. From the attacker’s perspective, Zhang *et al*. investigated how to design proper deception attack strategies in order to degrade the remote estimation quality [20]. They designed an online attack strategy and proved that the remote state estimation quality indeed becomes worse under this attack. For the energy-constrained DoS attacker, an optimal attack strategy has been presented to maximize the cost of LQG control [55]. The authors further studied the attack effect on the systems with multiple subsystems [56]. Qi *et al*. [57] considered the event-based attack strategy against remote state estimation. They provided an intelligent attack strategy that can destroy the estimation quality by leveraging the online measurement information. From the viewpoint of the attacker, they presented a simple form of the Minimum Mean Square Error (MMSE) estimation algorithm at the attacker side and a closed form of attack threshold, which can avoid the intrusion detection.

Different from traditional work on network security, the defender can design secure control laws to protect the control performances in CPS [58]. Yuan *et al*. [59] presented resilient controllers for CPS under DoS attacks. They proposed a coupled design framework of intrusion detection mechanisms and the robust control policy. Pasqualetti *et al*. [60] studied the distributed identification of attacks on a CPS. They modeled the state attack and the output attack as false data injection. Then, the distributed attack identification approach was proposed. This approach has the advantages of low computational complexity and performance guarantee. Ahmet *et al*. [61] investigated the event-triggered control against jamming attacks. They characterized the random packet losses and jamming-related packet losses and combined them together in the network. According to this characterization, they presented sufficient conditions for almost sure asymptotic stabilization and provided an approach to obtain the event-triggered controller.

### 2.5. Game-Theoretic Analysis

Game theory is a powerful tool for the investigation of CPS security. Typical approaches include zero-sum games [62,63,64], leader-follower games [65] and mixed games [66,67].

Gupta *et al*. [62] studied a dynamic zero-sum game between one controller for a plant with a discrete linear process and one malicious jammer whose objective was to block the communication between the controller and the physical plant. The jammer action is limited to a finite number due to the energy constraint over a finite horizon. They formulated the problem as an extended game and provided solutions to this problem for some special cases. A closed-form solution for general cases where the number of jamming actions is greater than one, however, is not presented due to the high computation complexity. Li *et al*. [63] also considered a DoS jamming attack for remote state estimation and control. Using channel hopping to avoid the jamming attack, a zero-sum stochastic game between the jammer and the sensor is formulated.

Using the leader-follower game, Langbort *et al*. [65] investigated the problem of one-step control over a communication network that introduces malicious packet drops. The communication network consists of several binary channels, which can be attacked by a jammer who has switching costs and constraints. The authors studied several types of games, including jammer leads and controller leads. They also provided closed-form solutions for scalar systems. For general higher order systems, however, closed-form solutions are difficult to obtain.

The mixed stochastic game was employed to investigate the security issue of CPS by Li *et al*. [66,67]. A sensor sends the data to the remote estimator via a wireless channel. The DoS attacker aims to destroy the estimation quality by jamming the communication medium. Unlike existing works, in the considered scenario, the attacker and the sensor both have energy constraints, and they formulated the security problem with a mixed game between the attacker and the system.

## 3. Cyber-Physical System Privacy

**Figure 3:**
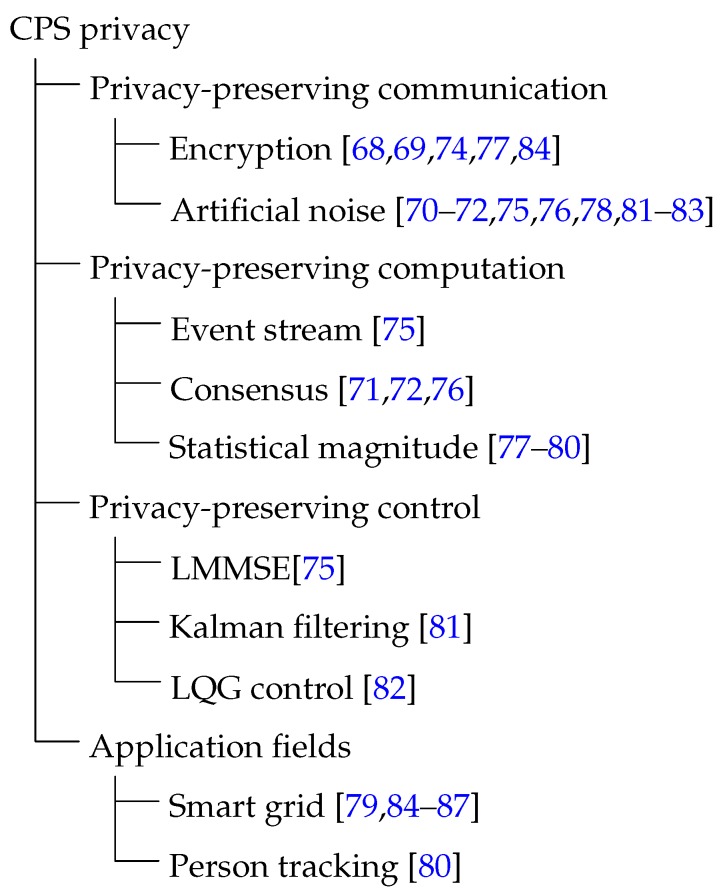
A summary of CPS privacy.

In this section, we discuss another important topic: CPS privacy. We will go over three main categories of works here. The first one in Section 3.1 introduces algorithms and designs to achieve private communication. The second one in Section 3.2 covers computation and control with privacy guarantee. The last one on privacy preserving in application fields will be introduced in Section 3.3. We also present a summary of CPS privacy in Figure 3 in the end of this section.

### 3.1. Privacy-Preserving Communication

Encryption and artificial noise injection are two main approaches for protecting individual privacy in the communication field.

A great deal of papers focused on designing encryption approaches to preserve the privacy in communication [68,69]. The seminal paper [68] first adopted an information-theoretic approach to study secrecy and privacy in a communication system. A transmitter broadcasts a message, which is encoded into a codeword, and two receivers (one being legitimate and the other being illegitimate) can receive the codeword. For discrete memoryless channels, the author showed that the perfect privacy preserving capacity is the difference between the respective mutual information. They further provided the requirements for a communication rate that has perfect secrecy. While [68] only considered discrete memoryless wire-tap channels, the authors in [69] extended the results to the Gaussian wire-tap channel. They gave the achievable region of the channel, which is determined by its secrecy capacity. The upper bound for the achievable rate-equivocation region was also established.

Artificial noise injection is another privacy-preserving approach for communication and has become a new hotspot recently. Goel and Negi [70] considered secret communication between one transmitter and one receiver over a wireless fading channel. A passive eavesdropper is present who can intercept the communication and obtain the communicated data. The transmitter, the receiver and the eavesdropper all have multiple antennas. The authors presented a strategy that adds artificial noise at the transmitter side. The added noise can be canceled by the receiver’s channel, but may not necessarily be canceled by the eavesdropper’s channel, thus potentially achieving secrecy. Manitara and Hadjicostis [71] considered average consensus algorithms and imposed a privacy constraint. In their proposed strategy, each agent adds a stochastic noise for a random number of periods and then subtracts the noise at the end of the random periods. By this strategy, the average consensus is not affected, and the initial state values are protected, e.g., no agent is able to infer the initial values of others. The authors also presented some topological conditions under which global malicious agents will not be able to infer initial values of agents. Mo and Murray [72] considered the same problem setup as in [71]. Through adding specially-designed Gaussian noise, their proposed method is able to protect the initial values while at the same time achieving consensus. Conditions for convergence and the convergence rate were provided. Akyol *et al*. [73] investigated the privacy-constrained communication from the game theoretic viewpoint. They aimed to minimize the mean square estimation distortion subject to the sender’s privacy constraint. The equilibrium conditions have been provided for compression and communication with the given privacy constraints. A significant feature of the provided solution is that it does not need the addition of independent noise. Artificial noise with a Laplace distribution was proposed to protect the location privacy of primary users and secondary users in cognitive radio networks [74].

### 3.2. Privacy-Preserving Computation and Control

From the viewpoint of computation, researchers have investigated how to design mechanisms for individual privacy preservation without destroying the computational performance, including event streams [75], consensus [71,72,76], statistical magnitude [77,78,79,80], *etc*.

An event stream problem with privacy requirements has been studied by Ny [75]. In the problem setup, a discretization input signal represents the appearance of certain events that are generated by customers, and a system sends some output signal based on the input signal while preserving the individual privacy. Differential privacy is adopted to guarantee the customers’ privacy requirements.

Huang *et al*. [76] studied private consensus, which requires the agents to protect the privacy of their initial values from a potential malicious adversary, who is able to obtain all messages being exchanged. Furthermore, convergence to the average of the initial states is required. The authors proposed a secure consensus algorithm with a differential privacy method, where the Laplacian noise is injected into each iteration during the consensus step. They showed that the randomized mechanism solves the synchronous private consensus problem and guarantees convergence to the average probabilistically. Tradeoffs between privacy and consensus accuracy were also discussed.

To obtain the sum statistics of multiple agents/sensors, secure data aggregation is utilized [77]. To ensure the privacy of each sensor, the author employed additive hemimorphic encryption and presented a novel key management technique to encrypt and decrypt the message between the sensors and the data aggregators. Computation of the sum of the initial values becomes straightforward using the encoded messages with the help of the key, while individual values are kept unknown. The algorithm can also be applied to min/max aggregation. He *et al*. [78] provided a new privacy protection approach, *i.e.*, Blowfish privacy, which can balance the utility of statistics and privacy in CPS. They presented novel algorithms that can handle the common privacy with physical constraints. They showed that Blowfish privacy can guarantee the performances in k-means clustering, histograms and range queries. Ny and Pappas [81] discussed mechanisms from a system-theoretic perspective to protect the individual privacy with differential privacy when the users transmit the data in a timely manner to a trusted central server. According to the inputs, the central server releases the cleansed aggregation outputs, which can protect the privacy from malicious adversaries.

When the eavesdroppers are present, the privacy requirements and control performances are both of vital importance for CPS. Zhang *et al*. [82] studied the trade-off between privacy preserving and system performance optimization from the viewpoint of the closed-loop in CPS. They formulated an optimization problem that maximizes the system control performance when the differential privacy requirement is given. For a linear system, the closed-form optimal system performance and corresponding controller are obtained under the desired privacy requirement. Ny [83] also investigated the privacy-preserving observer for the nonlinear process. The nonlinear estimation approach has been designed to observe the process with the differential privacy requirement. After theoretical research, the approach is applied to two examples, *i.e.*, an online social network with a dynamic stochastic block model and a real-time syndromic surveillance system.

### 3.3. Privacy Preserving in Application Fields

In the specific application fields, privacy is of great concern for people in a CPS. In a smart gird, the customer’s habits can be easily deduced from the real-time power consuming data [82]. Metke and Ekl [84] showed that the use of distributed intelligence and broadband communication so as to upgrade the current electric grid system requires new security technology. The authors discussed Public Key Infrastructure (PKI) technology, a key security technology, which they believe to be the best overall security solution for a smart grid. In order to prevent the electricity consumption data and electricity provider’s information from malicious attackers, Fan *et al*. [79] presented a novel data aggregation approach to enhance the privacy. Blinding factors are employed to generate the blinded data. From these data, only aggregation information can be obtained by the attackers. Tan *et al*. [85] provided a storage device system to enhance the customer’s privacy and the energy efficiency simultaneously. Liu *et al*. [86] provided a new load scheduling approach to minimize the total financial cost with the constraint of the physical devices and the individual privacy requirements of the customers. Sandberg *et al*. [87] constructed a framework to study the differential privacy mechanism in a smart grid. The customers are often unwilling to share their real-time usage data due to the privacy requirement. The proposed state estimation scheme can guarantee the customers’ privacy while optimizing the quality of estimation.

A real-time person tracking system is susceptible to personal privacy leakage. The time-of-flight images are significantly downsampled to protect the occupant’s privacy, as well as to simulate potential applications that exploit single-pixel sensors in the intelligent ceiling panels. Jia and Radke [80] presented a privacy-preserving tracking algorithms by reconstructing the image. They are also designed in a way to differentiate pieces of furniture from people. Based on the Markov stochastic model, a maximum likelihood estimation algorithm was designed for robust pose classification.

## 4. Conclusions

In the article, we have made a survey on the Security and privacy in CPS from the viewpoint of the closed-loop. We present the latest achievements in CPS security, including security analysis of general Cyber-Physical Systems, security issues in smart grid and power systems, secure algorithms, secure state estimation and control, game-theoretic analysis. The corresponding literatures is classified in Figure 2. Then the recent researches on CPS privacy have been discussed from the aspects of communication, computation, and control, respectively. Figure 3 provides a summary of the literatures on CPS privacy.

After introducing some recent works on CPS security and privacy, we would like to conclude this paper by discussing a few research directions that we think are important in the analysis and design of a secure CPS with privacy guarantee. We hope that these exciting research directions will trigger significant interest from many research communities and many profound results with deep impact will appear.

1. Modeling of CPSs: Currently, most models of CPS either focused on the physical side of the system (models from control theory) or focused on the cyber system (models from computer science). These models may not be sufficient to analyze the effects of cyber attacks on real physical systems. For example, an attack on cyber systems may cause a control algorithm to fail and cause the instability of the actual physical systems. On the other hand, an attack on the physical systems may cause a blackout and shut down the cyber systems. Furthermore, an adversary could even launch a hybrid attack on both physical and cyber systems. Hence, it is important to develop a unified framework and model to address the ever-increasing security concern in CPSs.
2. Modeling of the attacks: Most current research focused on an attacker that has full knowledge of the system. Such an attacker can be seen as the worst-case attacker. However, in practice, it is very difficult for an adversary to obtain such information. For example, in a smart grid, even the system operator does not have a full accurate model of the system, let alone the attacker. As is shown in [12], the first CPS malware, Stuxnet, only requires the attacker to know that the system is in a steady state. Therefore, assuming an adversary with full knowledge may lead to an over-conservative system design.
3. Measures of privacy: The existing studies of CPSs privacy are still in their infancy. In particular, we lack rich mathematical models of measuring CPS privacy. How to define proper privacy measures and how to design appropriate and corresponding algorithms to maximize these measures are important questions to be answered.
4. Joint design of intrusion detection, state estimation and control algorithm: As is illustrated in [12], the CPS can use noisy control to detect whether the sensory data have been replayed, with some loss of control performance. This illustrates the power of a jointly-designed estimation and control algorithm and intrusion detection algorithm. In other words, the estimation and control algorithm can excite the system in such a way that makes the attacker’s action visible to the intrusion detection algorithm. Consequently, the intrusion detection algorithm can detect malicious components and send alerts to the estimation and control algorithm. It is still quite an open problem how to find the optimal design.
5. Verification and validation: The control algorithm of a CPS could potentially be very complicated, which may involve thousands or tens of thousands lines of code. Hence, it is important to provide verification and validation tools to check whether potential security vulnerability may be introduced.
6. Secure atomic operation: Is it possible to build a secure algorithm from secure atomic operations? In software engineering, the defense against many different attacks is provided by the safe libraries. For example, the secure string library for C provides defense against buffer overflow attack. Therefore, the software engineers do not need to care about the buffer overflow attack as long as they use the safe library. In CPS, it would be great to define some secure atomic operations (for example, the median is more secure than the average) and to prove that any algorithm that only uses such secure operations will also be secure, so that a control engineer would not need to worry about security during his/her design process.
